# The individual- and community-level women's empowerment and utilization of maternity care services in Afghanistan: a multilevel cross-validation study

**DOI:** 10.1093/inthealth/ihad116

**Published:** 2023-12-21

**Authors:** Omid Dadras

**Affiliations:** Department of Global Public Health and Primary Care, University of Bergen (UiB), 5009 Bergen, Norway; Department of Addiction Medicine, Haukland University Hospital, 5012 Bergen, Norway

**Keywords:** Afghanistan, antenatal care, institutional delivery, maternity care, postnatal care, women's empowerment

## Abstract

**Background:**

This study aimed to explore the relationship between women's empowerment and utilization of maternity care for married Afghan women aged 15–49 y in Afghanistan, assessing the convergence validity of the Survey-based Women's Empowerment Index in Afghanistan (SWEI-A).

**Methods:**

The study used data from the 2015 Afghanistan Demographic Health Survey to examine the association of different domains of women's empowerment with the utilization of maternity care using multilevel Poisson regression at both individual and community levels.

**Results:**

The utilization of maternity services was considerably higher among women with high scores compared with those with low scores in almost all domains of the SWEI-A, except for property owning, in which women with high scores appeared to have lower rates of utilization of such services compared with those with low scores. At the community level, those communities with high participation of women in the labor force were less likely to have adequate antenatal care (ANC), institutional delivery and postnatal care (PNC). Individual-level literacy was associated with higher utilization of ANC, institutional delivery and PNC, contrary to community-level literacy.

**Conclusions:**

Except for property owning, the high score in almost all other domains was associated with higher utilization of maternity care, which indicates an acceptable level of convergence validity for the developed index (i.e. the SWEI-A) in measuring women's empowerment among married Afghan women aged 15–49 y.

## Introduction

Empowering is described as enabling vulnerable populations such as underprivileged women by eliminating the potential obstacles to using one's ability to make decisions and act independently, in which an individual’s well-being and integrity are granted.^1,2^ Women's empowerment is the key to achieving Sustainable Development Goal 5—achieving gender equality by 2030—and could be facilitated through equal rights and opportunities in terms of education, employment and healthcare for females compared with males.^3^ According to Kabeer, in one of the first definitions of women's empowerment, it is defined as: ‘agency’, indicating decision-making abilities regardless of the existing power structure; ‘resources’, which are described as channels through which one exercises agency such as education, health and physical assets; and ‘achievements’, which are the product of agency such as economic and sociopolitical gains.^2^ However, the multidimensionality of women's empowerment and diversity in context-specific sociodemographic indicators has raised several disputes around this definition and evidence suggests using context-specific scales to measure women's empowerment.^4,5^

Although there are several scales measuring women's empowerment, such as the Gender-based Development Index, the Gender-based Empowerment Measure and the Gender-Equality Index, the operational inadequacies in data and geographical coverage limit the use of such scales.^6^ Additionally, the availability of suggested indicators is often limited at the national level and is a disadvantage in poorly resourced countries such as Afghanistan where the available indicators are not genuinely representative of gender-based disparities.^7^ Therefore, defining reliable and context-specific variables is necessary for capturing the multidimensional structure of women's empowerment in a specific context.

Against this background, a Survey-based Women's Empowerment Index in Afghanistan (SWEI-A) was developed using the relevant indicators available in the latest Afghanistan Demographic Health Survey (ADHS) conducted in 2015. The primary analysis indicated satisfactory internal reliability (Cronbach's α=0.69) and construct validity (RMSEA&SRMR<0.05, CFI&TLI>0.95) in the primary analysis.^8^ To ensure the applicability of the index and comparability of the results across future studies, we decided to further measure the convergence validity of the SWEI-A by examining the differences in the utilization of maternity continuum of care across the domains of the developed index. Previous studies indicated a robust association between women's empowerment and utilization of reproductive and maternity healthcare such as modern contraception, antenatal care (ANC), institutional delivery, skilled birth attendance and postnatal care (PNC).^9–15^ However, the failure to use a unified index to measure women's empowerment limits the comparability of results across different settings. In addition, to the best of our knowledge, no study has ever reported on the impact that women's empowerment could have on the utilization of maternity care services in Afghanistan using a country-specific scale. Therefore, in this study, we aimed to explore the utilization of the maternity continuum of care including ANC, institutional delivery and PNC across the different domains of women's empowerment suggested by the SWEI-A, an index that was specifically developed to measure women's empowerment in married women aged 15–49 y in Afghanistan. Recognizing the evolving landscape for women's rights in Afghanistan following the recent changes by the Taliban Government, this study's focus on women's empowerment and its impact on maternity care gains further significance. Our exploration into the utilization of maternity services among married Afghan women aged 15–49 y, using the SWEI-A, takes place against a backdrop of dynamic sociopolitical changes that warrant acknowledgment. While our study predates these recent developments, our findings hold relevance in understanding the implications for women's access to healthcare services amidst evolving circumstances. We aim not only to shed light on the pivotal role of women's empowerment in enhancing maternity care utilization, but also to assess the applicability of our developed index (i.e. the SWEI-A) within the changing context of Afghanistan.

## Methods

### Study design

This study used data from the 2015 ADHS, which is the latest nationally representative survey implemented by the Central Statistics Organization in collaboration with the Afghanistan Ministry of Public Health and funded by the United States Agency for International Development.

### Study population and sampling

The 2015 ADHS collected data for women aged 15–49 y and their children aged <5 y through a stratified two-stage cluster sampling to estimate the key indicators at the national level, in urban and rural areas, as well as for each of the 34 provinces in Afghanistan. In the first stage, 950 clusters (enumeration areas from the previous national census), including 260 urban and 690 rural areas, were selected. In the second stage, through an equal probability systematic selection process, 25 650 households were selected within 950 clusters. To obtain representative estimates at the national level, sampling weights were calculated and applied. A sample of the women aged 15–49 y (n=29 641), who were either permanent residents of the selected households or visitors who stayed in the households the night before the survey, was recruited after providing informed consent. In this study, the analysis was restricted to married women aged 15–49 y because some questions related to the utilization of maternity care were only asked of married women.

### Study variables

A standard DHS questionnaire gathered data on sociodemographics, health status and access to health services from all eligible women aged 15–49 y. This includes women's demographic characteristics, household information, family planning, fertility preferences, maternity care, the health of women and children, sexually transmitted diseases, sexual behavior and domestic violence.^16^ The variables of interest in this study were those related to women's empowerment as suggested by the SWEI-A^8^ and those that addressed access to maternity care among married women aged 15–49 y in the 2015 ADHS.

### Independent variables

1. Women's empowerment: this was measured using the SWEI-A, which was specifically developed to measure the different dimensions of empowerment in married Afghan women aged 15–49 y. The SWEI-A measures women's empowerment in seven domains across four dimensions, as follows^8^:

- The economic dimension, which consists of two domains, namely, ‘labor force participation’ and ‘property owning’. Labor force participation included the following indicators: the respondent's occupation, type of earning from the respondent's work, seasonality of the respondent's occupation and work autonomy. Property owning was represented by legally owning a house or land variables.
- The sociocultural dimension, which consists of three domains, namely, ‘decision making’, ‘attitude toward violence’ and ‘age at critical life events’. Participation in decision making was assessed by three items: (i) the person who decides the respondent's healthcare; (ii) the person who decides on large household purchases; and (iii) the person who decides whether the respondent can visit her family or relatives. Attitudes towards violence were assessed using five variables describing whether beating was justified if the wife: (i) goes out without telling her husband; (ii) neglects the children; (iii) argues with her husband; (iv) refuses to have sex with her husband; and (v) burns food. The age at critical life events domain was measured by two indicators, consisting of age at first birth and age at first cohabitation.
- The education dimension, which has only one domain, namely, ‘literacy’, which was measured by two indicators: (i) the ability of the participants to read, and (ii) the highest educational level of the participant.
- The health dimension, which has only one domain, namely, ‘access to healthcare’, which was classified by four indicators examining the difficulty in obtaining medical help (not a big problem=1, a big problem=0), consisting of (i) receiving permission before obtaining medical help; (ii) having money to pay for healthcare; (iii) the distance to the health facility; and (iv) not wanting to go to the healthcare facility alone. Table 1 shows the details of variables related to women's empowerment selected in the current study, as suggested by the SWEI-A.

### Outcome variables

Three indicators related to the utilization of maternity services along the continuum of care were selected as outcome variables:

- ‘Adequate ANC’: based on the WHO recommendation, this is defined as having at least four ANC visits and is coded either as ‘yes=1’ for women with at least four ANC visits during their most recent pregnancy in the last 5 y, or as ‘no=0’ if there were fewer than four visits.
- ‘Institutional delivery’: this variable is coded either as ‘yes=1’ indicating delivery at health facilities, or as ‘no=0’ indicating delivery at home or elsewhere.
- ‘PNC’: this indicates whether the newborn received postnatal care within 2 mo (yes=1; no=0).

#### Data analysis

The relevant variables were extracted from the 2015 ADHS dataset and either recoded or retained in their original format for further analyses (Table 1). Descriptive statistics described the participants’ sociodemographic characteristics and the prevalence of the outcome variables (Table 2). The categories (low, medium, high) for women's empowerment domains were obtained by pooling the individual indicators’ scores and approximating the terciles as the cut-off points, as suggested by the SWEI-A.^8,10,17^ As presented in Table 3, we compared the women in the high tercile with the women in the low tercile (the reference group) in terms of the utilization of reproductive and maternity care services, to avoid masking the effect of women's empowerment by the scores in the middle tercile and highlighting the significant association between each construct and the outcome, as recommended by Ewerling et al.^10^ The convergence validity of the SWEI-A was measured by estimating the prevalence of outcome variables—namely, adequate ANC, institutional delivery and PNC—across different domains of women's empowerment at individual and community levels using multilevel Poisson regression analysis, as recommended by Barros and Hirakata.^18^ We defined communities as groups of respondents who share a common primary sampling unit within the DHS dataset based on the DHS statistic guides.^19^ All the analyses were adjusted for household wealth to assess the association of individual- and community-level women's empowerment indicators with the three outcomes of interest, independent from the household's wealth.^10^ The results were reported as prevalence rate ratio (PRR) and 95% CI. The sampling design and sampling weights were applied to account for the disproportional selection at urban and rural as well as cluster levels, and to obtain accurate and representative estimates at both national and regional levels. All the analyses were performed in STATA software version 16 (College Station, TX: StataCorp LLC) and p<0.05 was considered a significant statistical level.

**Table 1.** Dimensions (D1), domains (D2) and variables used in describing women's empowerment

D1	D2	Indicator	Questions	DHS response categories	Recode used in analysis
**Economic**	Labor force participation	Occupation	Type of work	Not working=0; professional/technical/managerial=1; clerical=2; sales=3; agricultural—self employed=4; services=7; skilled manual=8 unskilled manual=9	Not working=0; non-skilled (unskilled manual/clerical/services)=1; agriculture self-employed=2; skilled worker (professional/technical/managerial/ skilled manual)=3
		Earning	Type of earnings from respondent's work	Not paid=0, Cash only=1, Cash and in-kind=2, In-kind only=1	Not working=0, Not paid=1, In-kind only=2, Cash and in-kind only=3, Cash only=4
		Seasonality	Respondent employed all year/seasonal	All year=1, Seasonal=2, Occasional=3	Not working=0, Occasional=1, Seasonal=2, All year=3
		Work autonomy	Who do you work for?	Family member=1, Someone else=2, self-employed=3	Not working=0, Family member=1, Someone else=2, Self-employed=3
	Property owning	Land ownership	Owns land alone or jointly	Does not own=0, Alone only=1, Jointly only=2, Both alone and jointly=3	Does not own=0, Jointly only=1, Alone only=2, Both alone and jointly=3
		House ownership	Owns a house alone or jointly	Does not own=0, Alone only=1, Jointly only=2, Both alone and jointly=3	Does not own=0, Jointly only=1, Alone only=2, Both alone and jointly=3
**Sociocultural**	Household decision making	Women's health	Person who usually decides on respondent's healthcare	Respondent alone=1 Respondent and husband/partner=2 Husband/partner alone=4 Someone else=5 Other=6	Husband/partner alone/Someone else/Other=0, Respondent and husband/partner=1, Respondent alone=2
		Large household purchases	Person who usually decides on large household purchases		
		Visiting relatives/family	Person who usually decides on visits to family or relatives		
	Attitudes towards violence	Goes out without telling husband	Beating justified if wife goes out without telling husband	No=0, Yes=1, Don't know=8	No=1, Yes/Don't know =0
		Neglects children	Beating justified if wife neglects the children		
		Argues with husband	Beating justified if wife argues with husband		
		Refuses to have sex	Beating justified if wife refuses to have sex with husband		
		Burns food	Beating justified if wife burns the food		
	Age at critical life event	Age at first birth	Age of respondent at first birth	Age (in y)	No change
		Age at cohabitation	Age at first cohabitation	Age (in y)	No change
**Education**	Literacy	Literacy	Reading abilities	Cannot read at all=0, Able to read only parts of sentence=1, Able to read whole sentence=2	No change
		Educational level	Highest educational level	No education=0; Primary=1; Secondary=2; Higher=3	No change
**Health**	Access to healthcare	Permission	Getting permission to go	Not a big problem=2; Big problem=1	No change
		Money	Getting money needed for treatment		
		Distance	Distance to health facility		
		Going alone	Not wanting to go alone		

## Results

### Sociodemographic characteristics of married women aged 15-49 y, 2015 ADHS

As Table 2 shows, almost one-half of the women were aged <30 y (48.16%). The majority were illiterate (83.43%) and unemployed (87.14%). Less than one-quarter were living in urban areas. The Pashtun and Tajik ethnic groups were the major representatives in the sample.

**Table 2.** The sociodemographic characteristics of married women aged 15–49 y and their utilization of reproductive and maternity care services (2015 ADHS)

	n (weighted%)
Age group, y	
18–24	7398 (26.23)
25–29	6328 (21.93)
30–34	4398 (14.97)
35–39	4195 (15.49)
>40	5918 (21.38)
Education	
No education	24 488 (83.43)
Primary	1928 (7.87)
Secondary or higher	2245 (8.70)
Occupation	
Not working	25 008 (87.14)
Non-skilled worker	474 (1.09)
Agriculture	1316 (2.12)
Skilled worker	1841 (9.65)
Currently working	
No	25 666 (88.55)
Yes	2910 (11.45)
Place of living	
Urban	6815 (23.27)
Rural	21 846 (76.73)
Ethnicity	
Pashtun	12 232 (39.68)
Tajik	8686 (32.48)
Hazara	2658 (9.82)
Uzbek	1978 (11.01)
Turkmen	619 (3.02)
Nuristan	1180 (0.65)
Baloch	335 (0.61)
Pashai	503 (0.87)
Other	419 (1.87)
Wealth index	
Poor	11 838 (40.21)
Middle	6110 (20.07)
Rich	10 289 (39.72)
Adequate ANC	3175 (18.21)
Had institutional delivery	9715 (51.91)
Had PNC	4413 (24.93)

An equal proportion (approximately 40%) were in either poor or rich wealth index groups. Less than one-fifth had adequate ANC, almost one-half had delivered in a health institution and only one-quarter had a PNC in their most recent pregnancy.

### The association of individual-level women's empowerment indicators with the utilization of maternity services along the continuum of care

As Table 3 illustrates, the prevalence of having adequate ANC was significantly higher among women who have high scores on labor force participation (PRR=1.40; 95% CI 1.20 to 1.64), decision making (PRR=1.42; 95% CI 1.19 to 1.70), age at critical life events (PRR=1.21; 95% CI 1.08 to 1.35), literacy (PRR=1.47; 95% CI 1.34 to 1.60) and access to healthcare (PRR=1.35; 95% CI 1.20 to 1.51) compared with those with low scores. A similar trend was observed for institutional delivery: women with high scores on labor force participation (PRR=1.17; 95% CI 1.06 to 1.29), decision making (PRR=1.11; 95% CI 1.00 to 1.23), age at critical life events (PRR=1.12; 95% CI 1.04 to 1.19), literacy (PRR=1.27; 95% CI 1.20 to 1.34) and access to healthcare (PRR=1.13; 95% CI 1.05 to 1.21) were more likely to have adequate ANC compared with those with low scores. However, the prevalence of having adequate ANC was significantly lower among those with high scores in property owning (PRR=0.92; 95% CI 0.85 to 0.99) compared with those with low scores. For PNC, only those with high scores on labor force participation (PRR=1.20; 95% CI 1.04 to 1.39), literacy (PRR=1.10; 95% CI 1.01 to 1.19) and attitude toward violence (PRR=1.12; 95% CI 1.04 to 1.23) were more likely to have PNC within 2 mo after birth compared with those with low scores.

**Table 3.** The association between emerged domains and four reproductive and health care access indicators among married women aged 15–49 y in Pakistan (PDHS 2017–18)

		Adequate ANC	Institutional delivery	PNC
	Domains	PRR (95% CI)^a^	PRR (95% CI)^a^	PRR (95% CI)^a^
Economic	Labor force participation^†^			
	Individual level	1.40 (1.20–1.64)*	1.17 (1.06–1.29)*	1.20 (1.04–1.39)*
	Community level	0.12 (0.07–0.21)*	0.23 (0.18–0.30)*	0.65 (0.47–0.90)*
	Property owning^†^			
	Individual level	1.08 (0.97–1.19)	0.92 (0.85–0.99)*	1.07 (0.96–1.20)
	Community level	0.48 (0.40–0.58)*	0.89 (0.81–0.99)*	0.95 (0.82–1.10)
Sociocultural	Decision making^†^			
	Individual level	1.42 (1.19–1.70)*	1.11 (1.00–1.23)*	0.96 (0.80–1.15)
	Community level	1.82 (1.55–2.14)*	1.28 (1.17–1.41)*	1.16 (1.02–1.32)*
	Age at critical life events			
	Individual level	1.21 (1.08–1.35)*	1.12 (1.04–1.19)*	1.03 (0.94–1.13)
	Community level	0.94 (0.74–1.19)	1.21 (1.06–1.39)*	1.18 (0.97–1.43)
	Attitude toward violence^†^			
	Individual level	1.08 (1.00–1.19)*	1.01 (0.95–1.06)	1.10 (1.01–1.19)*
	Community level	1.24 (1.02–1.51)*	1.04 (0.93–1.16)	0.84 (0.72–1.00)
Education	Literacy^†^			
	Individual level	1.47 (1.34–1.60)*	1.27 (1.20–1.34)*	1.12 (1.04–1.23)*
	Community level	1.78 (0.92–3.43)	1.26 (0.85–1.88)	1.23 (0.69–2.20)
Health	Access to healthcare^†^			
	Individual level	1.35 (1.20–1.51)*	1.13 (1.05–1.21)*	1.04 (0.93–1.16)
	Community level	1.35 (1.19–1.51)*	1.12 (0.98–1.28)	0.68 (0.56–0.83)*

a PRR (95% CI): prevalence rate ratio and 95% confidence interval, adjusted for the wealth index.

*p<0.05.

^†^The high tertile was compared with the low tertile (reference group).

### The association of community-level women's empowerment indicators with the utilization of maternity services along the continuum of care

As Table 3 shows, the prevalence of having adequate ANC, institutional delivery and PNC is 88%, 77% and 35% lower, respectively, in those communities with high scores in labor force participation compared with those with low scores. In addition, communities with higher scores in property owning are less likely to have adequate ANC (PRR=0.48; 95% CI 0.40 to 0.58) and institutional delivery (PRR=0.89; 95% CI 0.81 to 0.99). The high-score communities in the decision-making domain were more likely to have adequate ANC (PRR=1.82; 95% CI 1.55 to 2.14), institutional delivery (PRR=1.28; 95% CI 1.17 to 1.41) and PNC (PRR=1.16; 95% CI 1.02 to 1.32). The communities with high scores on age at critical life events and attitude toward violence were, respectively, more likely to have institutional delivery (PRR=1.21; 95% CI 1.06 to 1.39) and adequate ANC (PRR=1.24; 95% CI 1.02 to 1.51) compared with those with low scores in these domains. In communities with high scores on access to healthcare, the prevalence of having adequate ANC was 1.35-fold higher and the prevalence of PNC was 32% lower compared with the communities with low scores.

## Discussion

This study assesses the utilization of maternity services along the continuum of care among Afghan women aged 15–49 y using a country-specific index, namely, the SWEI-A. The primary objective was to assess the convergence validity of the index, examining how well it can predict the utilization of maternity services, which are strongly associated with higher women's empowerment. The study examined the prevalence of utilization of maternity services along the continuum of maternity care including ANC, institutional delivery and PNC in relation to the seven domains of women's empowerment that are measured by the SWEI-A at both individual and community levels. The results showed that the rate of utilization of maternity services is considerably higher among those women with high scores in almost all domains, except for property owning, in which women with high scores appeared to have lower rates of utilization of such services compared with those with low scores. Although owning land or a house is linked to higher women's abilities in decision making in the household in several countries,^20,21^ in some Islamic states such as Afghanistan and Pakistan, particularly among those of low socioeconomic status living in poor families, receiving a piece of land or a house is considered to be a form of dowry that the bride’s family has received from the husband's family and, therefore, owning land or a house does not necessarily indicate a woman’s higher socioeconomic status, or indicate a translation into her having more power.^22^ This could act as a call for a re-evaluation of the developed index to assess whether discarding ‘property owning’ could improve the construct validity and reliability of the model.

Although at the individual level the rate of adequate ANC, institutional delivery and PNC appeared to be higher in women with high scores compared with those with low scores in the labor force participation domain, at the community level, those communities with a high participation of women in the labor force were less likely to have adequate ANC, institutional delivery and PNC. The underlying explanations provided for this contradictory finding with the individual- and community-level indicators have been inconsistent across previous studies. Some studies indicate that higher participation of women in the labor force could improve their empowerment through higher financial independence and decision-making ability^23–25^; however, most studies fail to report on the differential effect that could exist between individual- and community-level indicators. Besides, the socioeconomic development status of the country is an important factor that should be considered in the interpretation of these results. It has been shown that women's participation in the labor force could be an indicator of higher empowerment and gender equality in high-income countries; however, in low-income countries such as Afghanistan, this could be a sign of a household's lower socioeconomic and welfare status and, in fact, it is poverty that has pushed the woman into the labor market in order to contribute to the household income.^26^ Therefore, the improved financial and welfare circumstances that could be provided by women's participation in the labor force at the household level do not always translate into women having more power in decision making, and women's autonomy may not necessarily be improved by their participation in the labor market, particularly in a patriarchal society such as Afghanistan, where most of the decisions concerning a woman's health are made by her male partner.^27^ This could explain the lower utilization of maternity services in communities with high scores in this domain compared with those communities with low scores.

Several studies have shown that women's attitudes towards violence are associated with higher women's empowerment and decision-making abilities and led to better access to and utilization of ANC, PNC and institutional delivery.^28–31^ However, in our study, it was only associated with having adequate ANC at both individual and community levels and PNC at the individual level. Women with strong attitudes against spousal violence are less likely to tolerate domestic violence and be more involved in household decision making, including those decisions related to their reproductive and maternity care, which led to the higher utilization of maternity services observed in this study.^32^ Nevertheless, our findings showed no association between women's attitudes towards violence and institutional delivery. This could be attributed to the limited access and difficulties in reaching those institutions with delivery services in Afghanistan, and not merely women's willingness or decision to use such services.^33^

Age at critical life events, including age at first marriage and age at first birth, is an important predictor of pregnancy outcomes. It has been shown that women who marry and become pregnant at very young ages are more likely to experience negative reproductive and maternal outcomes, such as unmet needs for family planning, inadequate ANC, preterm labor, miscarriage, abortion, non-institutional delivery, postpartum hemorrhage and inadequate PNC.^27,34^ This has been attributed not only to the physical immaturity of young brides, but also to their low power in decision making and inability to negotiate their reproductive and maternal rights with their partner, which leads to lower access to and utilization of maternity care.^32,35^ Young brides are often the victims of spousal violence in patriarchal societies such as Afghanistan, in which women are considered to be inferior to men.^32^ The findings of this study reflect the impact that early marriage and conception can have on the utilization of maternity care, because women with high scores in the age at critical life events domain at both the individual and community levels were more likely to have adequate ANC and institutional delivery compared with those with low scores. However, there was no significant association between age at critical life events and PNC. The limitations in access to PNC aside, the low quality of such services is one of the discouraging factors in the utilization of PNC in poorly resourced settings such as Afghanistan.^36^ In addition, older mothers—those with high scores in the age at critical life events domain—often have more exposure to such services, and therefore low quality of PNC could be a stronger disappointing factor for this age group compared with younger age groups and this could further reduce the utilization of PNC by this age group; however, more studies are recommended to characterize the underlying drivers of such trends in the Afghan population.

Literacy plays a vital role in improving the majority of health outcomes.^37^ It has been shown that literate women are more likely to use maternal and reproductive services.^38^ Regardless of the number of years, women who can read experience better pregnancy outcomes and appear more frequently for their regular maternity visits.^39^ Literacy is also an important predictor of women's empowerment and higher education has been linked to higher decision-making abilities and more participation in the labor force, which are associated with higher women's empowerment.^10^ Nonetheless, our study showed that only individual-level literacy is associated with the utilization of maternity services. This emphasizes the important role of individual literacy rather than community literacy in the utilization of maternity care in a poorly resourced setting because individuals with higher literacy often have more health literacy and navigation skills to pursue healthcare, even if they live in a community with low literacy. However, amidst changing educational policies under the Taliban regime, the discussion should consider how altered literacy programs might impact women's autonomy and healthcare decision making.

Nevertheless, although the current study reported an acceptable level of convergence validity for the developed index (i.e. the SWEI-A) in estimating women's empowerment among married Afghan women aged 15–49 y, it is imperative to acknowledge the evolving landscape in Afghanistan. The recent shifts in governance have significantly impacted women's circumstances. The findings reflect a context that may have changed since the current study was conducted because of the evolving political situation after the Taliban; however, the implications of the findings for improving women's empowerment and health are undebatable. The SWEI-A remains vital in guiding future policies and interventions, despite the changing dynamics, offering a foundation for comprehensive studies and policy frameworks that aim at understanding and addressing the evolving needs of women in Afghanistan.

## Limitations

Some limitations should be considered in the interpretation of the results. First, the possibility of socially desirable responses due to the self-reported data could bias the estimates and distort the relationships between the explanatory and the outcome variables. Second, the cultural difference in perception of women's empowerment is not considered in the DHS; thus, the answers for some variables, particularly those addressing attitudes towards violence, may be biased. Third, the socioeconomic development of the country may influence the norms and cultural customs over time; therefore, periodical updates are crucial. Finally, most of the questions concerning women's empowerment were only asked of married women, while widows, single, divorced and separated women were excluded; therefore, the index is only applicable to married women in Afghanistan.

## Conclusions

The current study evidenced the acceptable convergence validity of the SWEI-A in assessing women's empowerment among married Afghan women aged 15–49 y concerning the utilization of the maternity care continuum. Notably, most domains, except for property owning, demonstrated a positive correlation with enhanced maternity care utilization. This prompts a reconsideration of the index's construct validity by possibly excluding the property-owning domain. Additionally, our findings underscore the nuanced impact of individual- vs community-level empowerment on maternity care utilization, particularly in resource-constrained environments. However, it is imperative to acknowledge that the sociopolitical landscape in Afghanistan, post-Taliban, presents evolving dynamics for women's conditions. Therefore, while our study provides valuable insights into women's empowerment and maternity care utilization, the changing context in Afghanistan’s post-Taliban regime requires ongoing scrutiny to comprehend its implications on women's empowerment and healthcare-seeking behaviors.

## Data Availability

The DHS questionnaire that collected the data in Pakistan's demographic and health survey in 2017–2018 can be downloaded from the DHS's official website (https://dhsprogram.com/data/available-datasets.cfm). The dataset (PDHS 2017-18) that was used in this study is available upon reasonable request and with permission from the DHS website.
